# The *PowerAtlas*: a power and sample size atlas for microarray experimental design and research

**DOI:** 10.1186/1471-2105-7-84

**Published:** 2006-02-22

**Authors:** Grier P Page, Jode W Edwards, Gary L Gadbury, Prashanth Yelisetti, Jelai Wang, Prinal Trivedi, David B Allison

**Affiliations:** 1 Section on Statistical Genetics, Department of Biostatistics, University of Alabama at Birmingham, AL, USA; 2 USDA ARS, Department of Agronomy, Iowa State University, Ames, IA, USA; 3 Department of Mathematics and Statistics, University of Missouri-Rolla, USA

## Abstract

**Background:**

Microarrays permit biologists to simultaneously measure the mRNA abundance of thousands of genes. An important issue facing investigators planning microarray experiments is how to estimate the sample size required for good statistical power. What is the projected sample size or number of replicate chips needed to address the multiple hypotheses with acceptable accuracy? Statistical methods exist for calculating power based upon a single hypothesis, using estimates of the variability in data from pilot studies. There is, however, a need for methods to estimate power and/or required sample sizes in situations where multiple hypotheses are being tested, such as in microarray experiments. In addition, investigators frequently do not have pilot data to estimate the sample sizes required for microarray studies.

**Results:**

To address this challenge, we have developed a Microrarray *PowerAtlas *[1]. The atlas enables estimation of statistical power by allowing investigators to appropriately plan studies by building upon previous studies that have similar experimental characteristics. Currently, there are sample sizes and power estimates based on 632 experiments from Gene Expression Omnibus (GEO). The *PowerAtlas *also permits investigators to upload their own pilot data and derive power and sample size estimates from these data. This resource will be updated regularly with new datasets from GEO and other databases such as The Nottingham Arabidopsis Stock Center (NASC).

**Conclusion:**

This resource provides a valuable tool for investigators who are planning efficient microarray studies and estimating required sample sizes.

## Background

Planning microarray studies provides unique challenges to investigators with respect to estimating power and the sample size required for a study. The questions proposed may be quite general and exploratory, such as "which genes are differentially expressed in response to a given treatment?" A microarray study should have a high probability to answer, at least in part, the questions and hypotheses being proposed [loosely speaking, power or in our case Expected Discovery Rate (EDR)]. It should also have a high probability that those genes declared significant are truly differentially expressed (i.e. the 'True Positive' probability should be high). Sample size is a critical determinant of statistical power and expected error rates.

In traditional biomedical studies, investigators test one or at most a few hypotheses. This is not the case in microarray studies. Each treatment or group comparison involves the testing of every gene on the chip, which may number in the 10,000's. Some microarray experiments may involve multiple groups; thus the total number of hypotheses tested in a microarray experiment can run in the 100,000 s or more. In addition, the effects size and variance for each hypothesis may be different; resulting in different power estimates for each and every gene by treatment comparison.

Some investigators have proposed approaches to estimating required sample size for microarray research [2-4], but most of these methods calculate power based upon an arbitrary level of change being biologically relevant and constant across all genes. These methods do not take into account the amount of variability in each gene nor specify a hypothesized distribution of effect sizes, and do not incorporate some of the recently developed approaches to account for multiple testing in high-dimensional biology(HDB) [5].

Calculating required sample sizes for a study requires an estimate of the variability in the dependent variable, in the case of microarray studies the genes' expression levels. This information is frequently derived from previous pilot studies performed by the research team or from similar data in the literature. Integrating information from pilot studies that illustrate the variability of all the genes in a study provides empirically driven and theoretically defensible sample size estimates. We have formalized such an approach [5]. Rather than using traditional power (1-β) we introduced the concept of *Expected Discovery Rate *(EDR). EDR is the average power (see table 1 for a definition of A, B, C, and D) for all genes for which the null hypothesis is false in an experiment. EDR is the E [Q] where Q = D/(D+B) if D+B>0 and Q = 0 otherwise and can be interpreted as the expected proportion of genes that are truly differentially expressed that will be declared to be differentially expressed. Microarray studies are affected by multiple testing issues; thus, when considering power studies one must not only consider the alpha (significance) level cut-off used, but also the expected proportion of genes that are True Positives (PTP), which is similar in concept to the False Discovery Rate. The PTP may be defined as (again A, B, C and D are defined in table 1) the E [R] where R = D/(C+D) if C+D>0 and R = 0 otherwise. The PTP is the expected proportion of genes that are declared significantly differentially expressed between the two samples that are actually differentially expressed between the two populations. A higher value for PTP is considered more desirable. The software also provides the *Probability of a True Negative *(PTN), which is the expected proportion of genes that are *not *declared significantly differentially expressed between the two samples that are actually not differentially expressed between the two populations. The use of EDR, PTP, and α provides a coherent way to estimate the sample size required for microarray research that is consistent with current approaches to analyzing microarray data and conceptualizing the process of massive multiple hypothesis testing in HDB research [6,7]. The *PowerAtlas *implements the methods developed by Gadbury et al [5] and adds further functionality.

**Table 1:** Quantities of interest in microarray experiments

	Genes for which there is not a real effect	Genes for which there is not a real effect
Genes not declared significant at designated thereshold	A	B
Genes declared significant at designated thereshold	C	D

The *PowerAtlas *works in two ways. Firstly, investigators may upload their own pilot data and extrapolate out the EDR, PTN, and PTP for a variety of sample sizes and α (type 1 error rate) level combinations. Secondly, many investigators do not have the opportunity to conduct their own pilot microarray study, but this need not stop an investigator. Given that many journals now require authors to place microarray data in public databases [8] before publication, investigators may draw upon these public data as pilot information. We have developed the *PowerAtlas *to assist investigators in the use of these public data to estimate the sample sizes required for well-powered studies. We have downloaded all data from Gene Expression Omnibus [9], reanalyzed it, and processed it with the methods developed in Gadbury et al [5]. Thereafter, we have put the power and sample size calculations for many of the datasets into a readily accessible and searchable database [1]. It should be stressed that no one study is a perfect replicate of the study an investigator wishes to conduct, but similar studies can give a sense of the plausible ranges of sample sizes. We recommend that investigators examine several related experiments to get a sense of the sample size required for robust EDR and high PTP.

Designing a microarray study with the appropriate number of replicates is cost efficient. The use of the *PowerAtlas *will not only prevent investigators from using too many samples in a group, resulting in wasted money; but will also limit wasting money on experiments that have too few replicates to have sufficient power to yield good results.

### Usage of the PowerAtlas

No registration is required to use the *PowerAtlas*, nor are any programs or applets pushed to an investigator's computer. An investigator simply accesses the *PowerAtlas *[1] and selects the appropriate link to use public data or the investigator's study-specific data.

The data in the *PowerAtlas *are taken directly from GEO. As long as it meets the requirements outlined in 'Using Existing Public Data' the data is included in the *PowerAtlas*. However, the data in GEO can be quite variable, due to any number of reasons, including, but not limited to, the image processing algorithm, normalization, and inferential statistical procedure used in the analysis. Thus when using public data as a basis for planning future studies, an investigator should consider the results from several datasets, consult the primary sources (GEO GDS files and journal publications of the data), and have a reasonable understanding of the idiosyncrasies and applicability of each dataset to the proposed experiment before using the data. In addition, since each lab processes and handles samples and runs microarrays slightly differently, when possible, estimates of power should be based upon an investigator's own pilot data, which will be more accurate for an investigator's future experimental power than will extrapolations from other investigators' data.

### Using the investigators' own data

**Figure 1:**
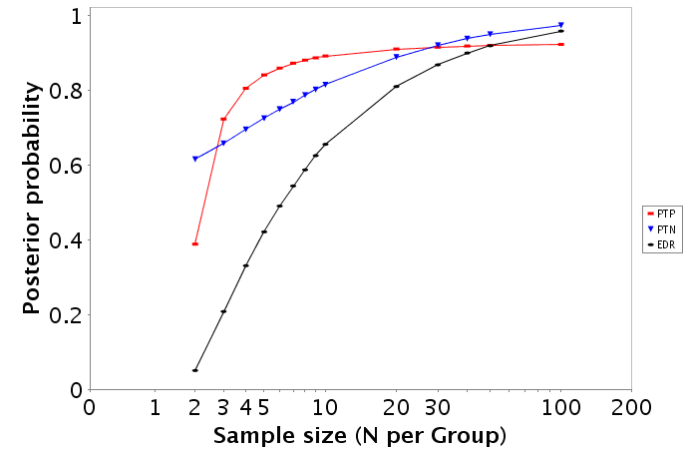
Estimated PTP, PTN, and EDR for the GDS486 [17] dataset for a variety of samples sizes at an alpha level of 0.05.

**Figure 2:**
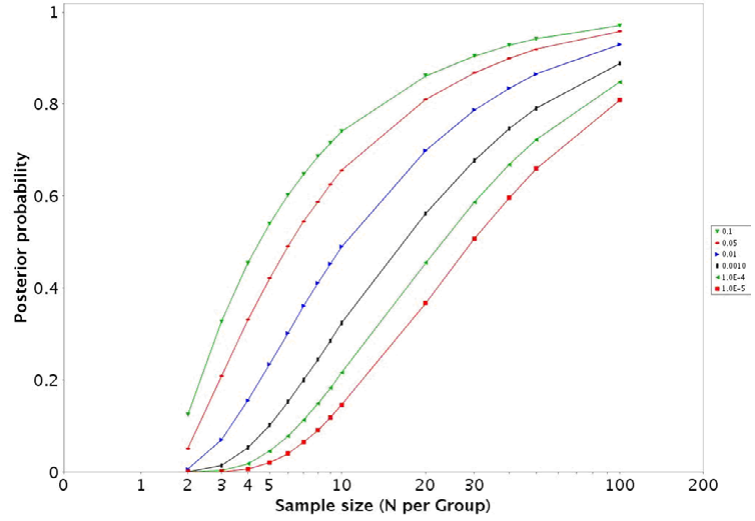
For the GDS486 dataset [18] the EDR is presented across a variety of sample sizes and alpha levels.

**Figure 3:**
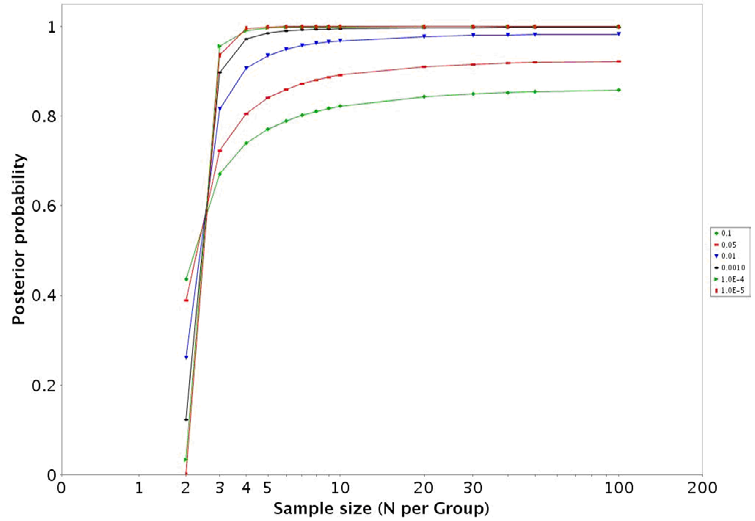
For the GDS486 dataset [19] the PTP is presented across a variety of sample sizes and alpha levels.

**Figure 4:**
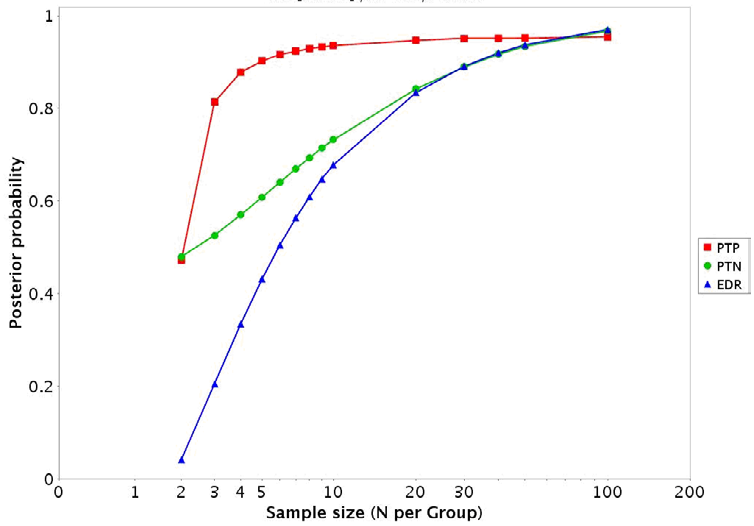
Estimated PTP, PTN, and EDR for the GDS75 [20] dataset for a variety of samples sizes at an alpha level of 0.05.

**Figure 5:**
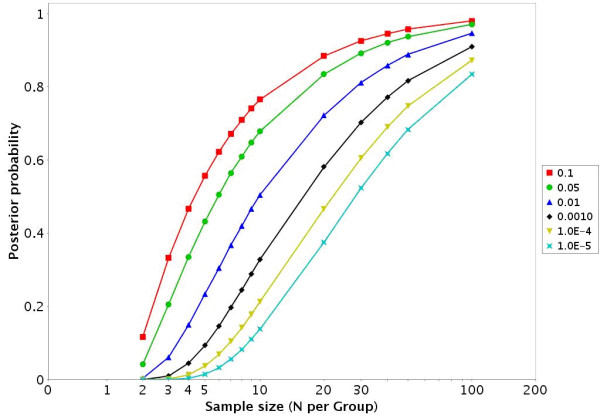
For the GDS75 dataset [21] the EDR is presented across a variety of sample sizes and alpha levels.

**Figure 6:**
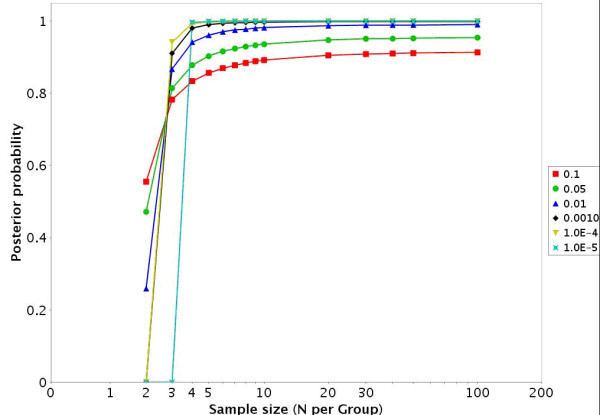
For the GDS75 dataset [22] the PTP is presented across a variety of sample sizes and alpha levels.

To use the *PowerAtlas *with an investigator's own data a list of p-values generated using a valid statistical method must be available. Currently the *PowerAtlas *generates sample sizes for two group comparisons only for any valid statistical test [10]. Then use the following instructions:

• The investigator must possess/generate a tab delimited file with one p-value per gene/feature for the main hypothesis of interest with each p-value located on its own line. There should be no identifiers for genes. All p-values from all genes on a chip/array should be included.

• The file with p-values is uploaded to the web site.

• The investigator then enters the sample sizes (N1 and N2) for each of the groups used to calculate the p-values.

• The investigator then may either use default or custom settings for the sample sizes, significance (α) thresholds, and number of iterations for the bootstrap to be used for estimating power.

• The investigator selects submit. For a sense of runtime, from an initial set of 12,500 p-values with EDR, PTP, and PTN being calculated for 14 sample sizes and six thresholds, the analysis takes 3–10 minutes.

• The investigator then will obtain a series of figures that illustrate the EDR, PTP, and PTN for a variety of sample sizes and significance (α) thresholds(examples are shown in figures 1, 2, 3 for an Affymetrix dataset [11] and figures 4, 5, 6 for a cDNA experiment [12]). The investigator may then choose the sample size and α level combination that achieves the desired levels for EDR, PTP, and PTN.

### Using existing public data

**Figure 7:**
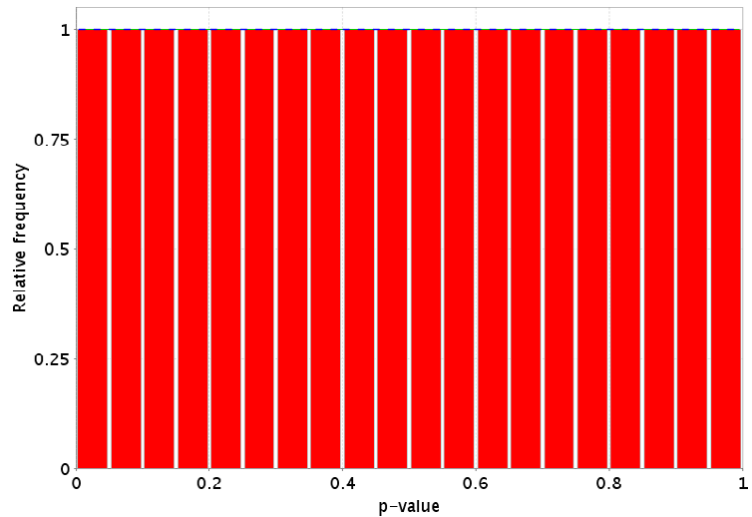
Idealized representation of the distribution of p-values under the null hypothesis (no difference in gene expression between the two groups) for a valid test. The dotted Blue line is the expected distribution of p-values if the treatment has no effect and the solid Green line is the mixed-model [23] fit of the p-value constrained to be monotonically non-decreasing.

**Figure 8:**
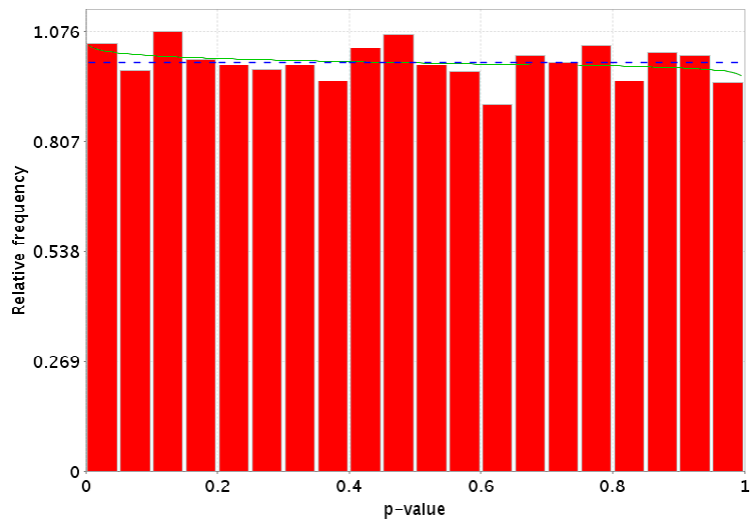
More realistic representation of the distribution of p-values under the null hypothesis (no difference in gene expression between the two groups) for a valid test. The dotted Blue line is the expected distribution of p-values if the treatment has no effect and the solid Green line is the mixed-model [23] fit of the p-value constrained to be monotonically non-decreasing.

**Figure 9:**
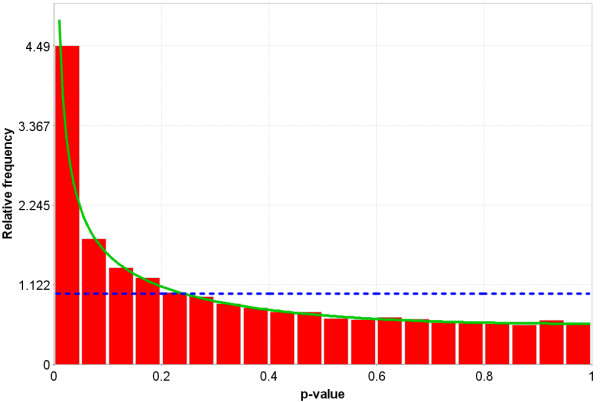
Distribution of p-values when there is a difference in the gene expression between the two groups for some of the genes, but not all of the genes. This distribution is monotonically non-increasing from 0 to 1. The dotted Blue line is the expected distribution of p-values if the treatment has no effect and the solid Green line is the mixed-model [23] fit of the p-value constrained to be monotonically non-decreasing.

**Figure 10:**
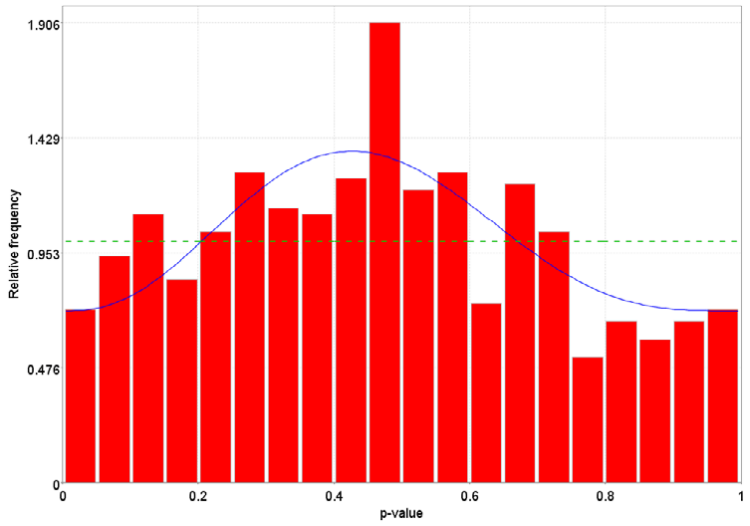
Distribution of p-values for a dataset in GEO that does not follow one of the possible distributions for p-values The dotted Blue line is the expected distribution of p-values if the treatment has no effect and the solid Green line is the mixed-model [23] fit of the p-value constrained to be monotonically non-decreasing.

**Figure 11:**
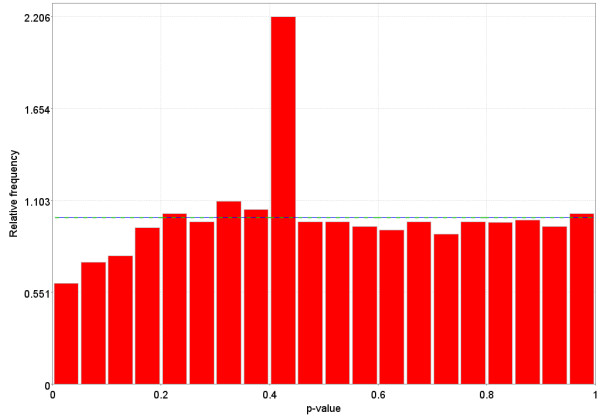
Distribution of p-values for a dataset in GEO that does not follow one of the possible distributions for p-values. The dotted Blue line is the expected distribution of p-values if the treatment has no effect and the solid Green line is the mixed-model [23] fit of the p-value constrained to be monotonically non-decreasing. The dotted Blue line is the expected distribution of p-values if the treatment has no effect and the solid Green line is the mixed-model [23] fit of the p-value constrained to be monotonically non-decreasing.

**Figure 12:**
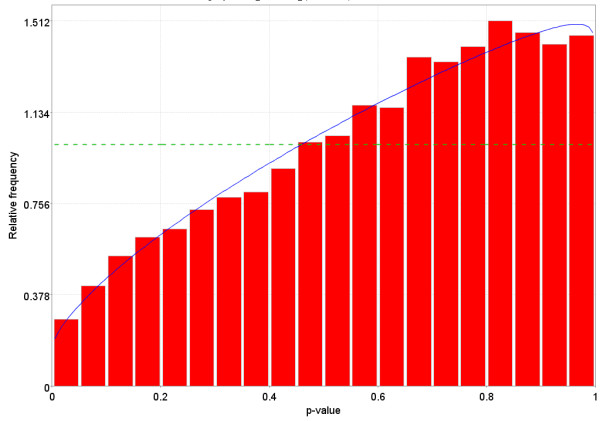
Distribution of p-values for a dataset in GEO that does not follow one of the possible distributions for p-values. The dotted Blue line is the expected distribution of p-values if the treatment has no effect and the solid Green line is the mixed-model [23] fit of the p-value constrained to be monotonically non-decreasing.

We have downloaded and processed all data that was in GEO's ftp site as of Oct 1, 2004 into the *PowerAtlas*. One type of information GEO allows to be entered is the group to which a chip belongs. We conducted a pooled variance t-test on all possible two-group intraGDS (GDS is the GEO definition of an experiment) comparisons from all datasets within GEO. During analysis some datasets were removed. Reasons for removal include: A) The data per chip was incomplete, for example, due to masking or selecting genes based on present or absent calls. B) The p-values did not follow the expected possible distributions (monotonically non-decreasing from 0 to 1). Figure 7 and 8 illustrate a null data set (no more genes are significant than are expected at random). Figure 9 illustrates a typical p-value distribution for a good dataset for which the null is false for some, but not all genes. Figures 10, 11, 12 represent distributions of p-values seen while processing GEO that do not fit the expected distributions and thus would be listed as NA. C) Only two group fully randomized cDNA experiments can be analyzed, which prevents some cDNA experiments, including all loop designs and those that involve dye swaps unless in a balanced block design from being analyzed. [13-15]. D) Datasets having fewer than two experimental groups and three chips per group are also excluded.

To use the public data for estimating sample size:

• From the *PowerAtlas *web page an investigator selects the option of using existing public data.

• The investigator makes a selection of desired chip type (one or two channel) and the species of interest. At most, one of each chip type or species of interest may be selected. Alternatively, the investigator may also select only a chip type or a species.

• A list of all experiments will appear that meet the selection criteria. The number of datasets can range from 0 (most bacteria on single channel chips) to more 200 for Human and Mouse on single channel chips (see table 2).

**Table 2:** Itemization of the number type and species of chips available. NA means power estimation is not available. See section "Using existing public data" for possible explanations why the datasets may have be listed as NA.

	Dual Channel	Dual Channel	Single Channel	Single Channel
	Available	NA	Available	NA
*Arabidopsis thaliana*	1	10	22	0
*Aspergillus parasiticus*	0	1	0	0
*Bacillus anthracis*	0	2	0	0
*Bos taurus*	0	3	2	0
*Caenorhabditis elegans*	1	2	1	0
*Campylobacter jejuni*	1	1	0	0
*Canis familiaris*	0	0	1	1
*Capra hircus*	0	0	1	0
*Chlamydomonas reinhardtii*	1	0	0	0
*Cricetulus griseus*	0	1	0	0
*Drosophila melanogaster*	3	9	15	0
*Drosophila simulans*	2	0	0	0
*Drosophila yakuba*	0	2	0	0
*Escherichia coli*	5	1	2	0
*Escherichia coli K12*	0	0	1	0
*Fundulus heteroclitus*	0	0	1	0
*Homo sapiens*	44	34	178	35
*Marmota monax*	0	0	1	1
*Mastomys natalensis*	0	0	0	1
*Mus musculus*	63	14	175	58
*Mycobacterium tuberculosis*	1	0	0	0
*Oncorhynchus mykiss*	0	4	0	0
*Oryza sativa*	0	2	0	0
*Pinus contorta*	0	0	1	0
*Rattus norvegicus*	6	8	68	13
*Rhodobacter sphaeroides*	0	0	2	2
*Saccharomyces cerevisiae*	12	41	18	1
*Saccharomyces pastorianus*	0	0	0	1
*Saccharum sp*.	0	0	0	1
*Salmo salar*	0	2	0	0
*Salmonella enterica*	1	0	0	0
*Sus scrofa*	0	1	1	0
*Viruses*	0	0	0	1
*Zea mays*	1	0	0	0

TOTAL	142	138	490	115

• The investigator can read a brief description, taken directly from GEO, of all the experiments and find those that are most similar to their proposed experiment(s).

• The investigator then selects the checkbox to the left of the desired datasets and press Submit to get additional information.

• The investigator receives a report with a link to the GEO description should additional information be needed.

• There are also links to a printable HTML report that includes a description of the dataset, the EDR, PTP, and PTN (figures 1 and 4) at an α level of 0.05 as well as a description of how to interpret the results.

• In datasets with more than 2 groups the two groups with the largest sample sizes are given in the HTML report. There is a link to jpeg images for the other IntraGDS comparisons in the data set. In addition there is a link to a downloadable zip file that contains graphs illustrating the EDR (figures 2 and 5), PTP (figures 3 and 6), and PTN for a variety of α and sample sizes in a directory structure for each two group comparison. There is also an Excel file provided that contains the numbers underlying the figures.

### Illustrative example of the accuracy and utility of the PowerAtlas

**Figure 13:**
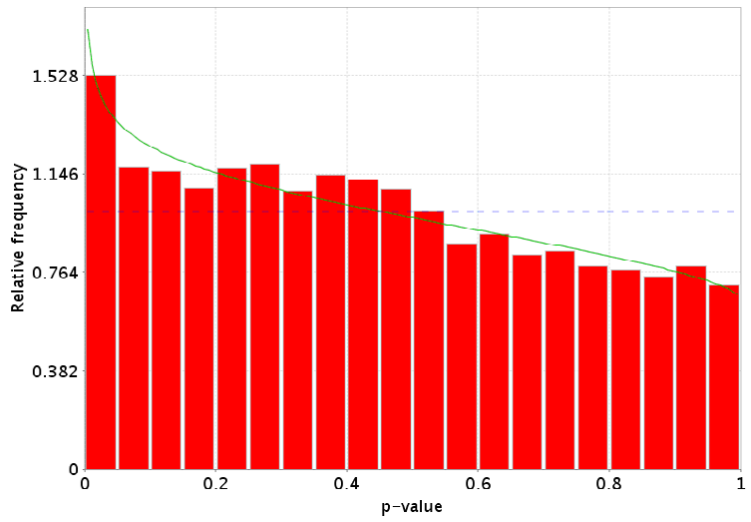
Distribution of p-values for the comparison of RNA from 3 murine with homozygous PKDH mutations and a high kidney length-to-width ratio and RNA from 3 mice with homozygous PKDH mutations and a low kidney length-to-width ratios. The dotted Blue line is the expected distribution of p-values if the treatment has no effect and the solid Green line is the mixed-model [23] fit of the p-value constrained to be monotonically non-decreasing.

**Figure 14:**
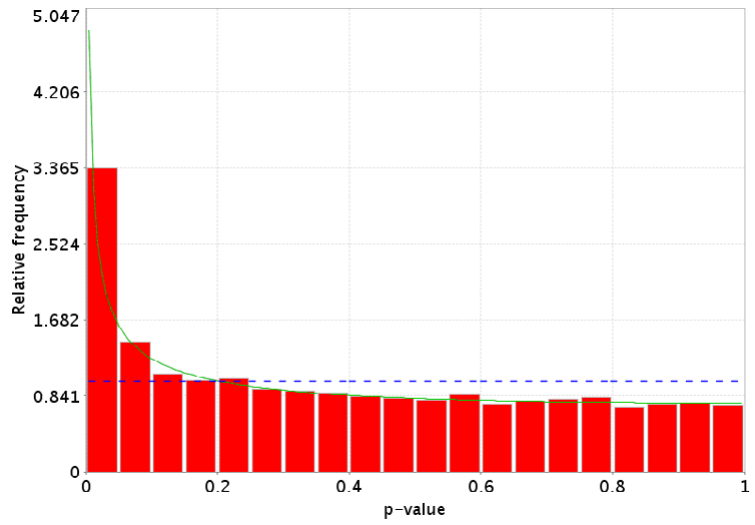
Distribution of p-values for the comparison of RNA from 7 mice with PKDH mutations and a high kidney length to width ratio and RNA from 7 mice with PKDH mutations and a low kidney length to width ratio. The dotted blue line is the expected distribution of p-values if the treatment has no effect and the solid green line is the mixed-model [23] fit of the p-value constrained to be monotonically non-decreasing.

We provide a concrete example for illustrative purposes. In one study (unpublished) the RNA kidneys from individual mice that were homozygous for a PKDPH mutation were collected. Mice were selected from a F2 cross that had a very high kidney length to width ratio (three mice) or a very low length to width ratio (three mice). This measure is one determinant of polycystic kidney disease severity. The RNA was run on the Affymetrix Mu74Av2 array and processed with MAS 5.0 (Affymetrix, Inc, Emoryville, CA). Figure 13 illustrates the distribution of p-values for the 2-group comparison of the gene expression levels between the high and low kidney length-to-width ratio mice. These p-values were run through the *PowerAtlas*. We selected a target sample size of seven per group to have an EDR of > 40% and PTP > 80% at α<0.05. An additional seven mice were run from each extreme of the kidney length-to-width ratio distribution. The distribution of the p-values for the seven mice per group comparison is given in figure 14. Table 3 illustrates the EDR and PTP at α = 0.05 and 0.0001 for a sample size of seven per group that were estimated from the initial sample of three per group (row 2). These numbers are compared to the actual EDR and PTP that were calculated at α = 0.05 and 0.0001 for the follow on study at a sample size of seven per group (row 3). The numbers are remarkably similar.

**Table 3:** Estimated EDR and PTP for sample size of 7 per group at alpha levels of 0.05 and 0.001 extrapolated from a sample size of 3 (row 2) from the PKD data and the estimated EDR and PTP group at alpha levels of 0.05 and 0.001 calculated in the follow up study of 7 mice per group.

	**Estimated EDR for SS 7** ** at α = 0.05**	**Estimated PTP for SS 7** ** at α = 0.05**	**Estimated EDR for SS 7** ** at α = 0.001**	**Estimated PTP for SS 7** ** at α = 0.001**
Pilot of 3 per group	0.415009	0.809616	0.119791	0.985287
Experiment of 7 per group	0.538771	0.772419	0.133711	0.976347

## Conclusion

The *PowerAtlas *provides investigators the option of using their own pilot data or drawing from a public domain microarray data sets to calculate sample sizes and statistical power for a proposed study. The overall goal is to estimate the sample size required to be able to answer the hypothesis of interest with a high EDR and a high PTP without using too many chips. Once the graphs and tables most appropriate are identified (which may involve examining several datasets), the investigator must decide upon the sample size to pursue. Unlike single hypothesis-driven research, a huge number of genes often are typically differentially expressed in a single microarray experiment and a study may yield many (often thousands) of significant genes. It is generally difficult for a single laboratory to follow-up or to investigate more than a few genes. Thus, while an EDR of 80% or more may be in line with traditional power studies, investigators may not want or have the laboratory resources to deal with large-scale high-powered gene expression experiments where 1000 s of genes are identified as differentially expressed. Thus, it may be more appropriate to have a small list of genes in which an investigator has high confidence that the genes identified as differentially expressed are truly differentially expressed. Thus, modest EDRs (10–40%) may be appropriate when conservative alphas are chosen to generate high PTPs (80%+). On the other hand, when the investigator wants to get a complete picture of the experimental manipulations it may be more appropriate to use a liberal alpha level (0.1) to have a high EDR, but this will yield a lower PTP. Investigators should carefully consider what error rate (the proportion of the genes that are studied further that are false positives) is acceptable, how many genes they can truly invest in studying, and how important it is to have a complete list of differentially expressed genes.

A few other issues should be considered when choosing the sample size for an experiment. The first, one should not rely upon any one study to drive the sample size. An investigator should view several datasets to get an idea of the range of possible sample sizes. Secondly, we have analyzed all the data in the *PowerAtlas *as if it were two groups with fully randomized designs. This may not be the case; experiments may be 2 or 3 way experiment with multiple levels. If the actual experiment were these designs, the calculated sample size may be an over estimate for the methods in the *PowerAtlas *does not yet allow for using information from other groups to estimate the variances as methods such as ANOVA and linear models do. In addition the hypothesis shown in the main graph may not be the primary hypothesis of interest in a study, they are simply the groups with the largest sample sizes, and the other hypotheses should be reviewed as well. Investigators should review the primary literature to verify what the true experimental design was. We also assume the experiments were conducted in a rigorous fashion and have not been confounded by non-biological sources of error, which may adversely effect power nor does the use of good sample size obviate the need for good experimental design and conduct of the experiment [16].

### Future directions

There are several areas where the functions of the *PowerAtlas *will be expanded. First we will augment the data in the database by revising the data from GEO every six months and we will add data from additional sources such as the Nottingham Arabidopsis Stock Center (NASC). In the PTP graph at low n and small α, the lines of the PTP sometimes cross, which is due to the fact under these conditions sometimes very few or even zero genes are declared significant. As this is the denominator of PTP the PTP is 0. Until the sample size gets large enough to declare enough genes differentially expressed at a chosen (small) threshold, PTP lines may cross over each other. We are working to eliminate this issue from our method. Currently, only point estimates of the EDR, PTP and PTN are generated. Future work will generate confidence intervals on these estimates. We are also extending the power estimation procedures to handle ANOVA and linear models, which will allow for power estimation for loops designs, the correct analysis of datasets with multiple groups, and time series data. When these methods have been developed they will be incorporated into the *PowerAtlas*.

## Availability and requirements

• **Project name: **The PowerAtlas

• **Project home page: **http://www.powerAtlas.org also http://www.poweratlas.net 

• **Operating system(s): **Web-based application

• **Programming language: **Java

• **Other requirements: **Web browser. Unzip utility

• **License: **None

• **Any restrictions to use by non-academics: **None

## Abbreviations

ANOVA: Analysis of Variance

EDR: Expected discovery rate

GDS: Gene Expression Omnibus Dataset

GEO: Gene Expression Omnibus

HDB: High Dimensional Biology

PTP: Probability of a True Positive

PTN: Probability of a True Negative

## Authors' contributions

GPP co-developed the concept of the *PowerAtlas*, co-developed the underlying statistics for the *PowerAtlas*, led the development of the *PowerAtlas *tool, and drafted the manuscript, JWE co-developed the concept of the *PowerAtlas*, DBA co-developed the concept of the *PowerAtlas *and co-developed the underlying statistics for the *PowerAtlas*. GLG Co-developed the underlying statistics for the *PowerAtlas*. PY, JW, and PT are software and database developers of the *PowerAtlas *tool and web-site.
